# Consanguineous Marriage: Law and Public Health

**DOI:** 10.1007/s10728-025-00541-2

**Published:** 2025-10-10

**Authors:** Nicola Glover-Thomas

**Affiliations:** University of Manchester, Manchester, UK

**Keywords:** Consanguineous marriage, Genetic health risks, Public health policy, Human rights, Marriage (prohibited degrees of relationship) bill 2025

## Abstract

This article examines the complex interplay of cultural practices, genetic health risks, and evolving legal frameworks surrounding consanguineous marriage, with a focus on England and Wales. Consanguineous unions, increase offspring’s risk of autosomal recessive genetic disorders and congenital anomalies due to heightened homozygosity. The ‘Born in Bradford’ study revealed that 37% of babies in the cohort were born to related parents, with over 60% of marriages in the Pakistani-origin population being consanguineous. This was associated with a near doubling of the congenital anomaly risk (3% to 6%) and accounted for an estimated 30% of all congenital anomalies and 25% of infant mortality in Bradford. While Norway and Sweden have recently banned or are set to ban first-cousin marriages, citing public health and forced marriage concerns, England and Wales maintain a permissive stance. Proposed legislation, like the Marriage (Prohibited Degrees of Relationship) Bill 2025, aims to prohibit these unions and is argued to mitigate NHS strain. However, such proposals face significant human rights challenges (right to marry, privacy, non-discrimination) and concerns about driving the practice underground. The paper concludes by advocating for nuanced, culturally sensitive public health strategies—including enhanced genetic counselling, targeted education, and proactive screening—to empower informed choice and improve health outcomes without legal coercion, aiming to balance state responsibility with individual and cultural freedoms.

## Introduction

The practice of consanguineous marriage, defined as unions between individuals who are second cousins or closer, remains a globally prevalent phenomenon, deeply embedded in the cultural and social fabric of numerous societies. While historically common across many civilisations, including European royalty, its contemporary prevalence is often concentrated in specific geographic regions and diaspora communities, particularly in parts of the Middle East, North Africa, and South Asia. This practice, while culturally significant for many, has increasingly become a subject of intense debate in Western societies, where discussions often intersect with concerns about public health, genetic risk, individual autonomy, and potential coercion, including forced marriage, and the state’s role in regulating private life. The sensitivity of this topic is heightened by its cultural dimensions, often leading to cautious approaches from policymakers and healthcare providers. Consequently, discussions and policy interventions must meticulously navigate these ingrained cultural questions to prevent offense, misunderstanding, or the alienation of the communities concerned.

Consanguinity, at its core, refers to the genetic relatedness between individuals. In a consanguineous marriage, the shared ancestry between spouses increases the likelihood of their offspring inheriting two copies of the same recessive allele from a common ancestor. This elevated homozygosity is the biological mechanism through which such unions are associated with an increased risk of specific genetic disorders and congenital anomalies [6].

This article will explore the complex interplay of cultural practices, genetic health risks, societal impacts, and the evolving legal and policy debates surrounding consanguineous marriages. While the primary focus of this article is marriage legislation, marriage itself is not a prerequisite for reproduction or for the legal recognition of parental status in many jurisdictions, including England and Wales. This nuance underscores the broader context of family formation beyond formal marital unions. With a particular focus on the context in England and Wales, the paper will draw insights from the landmark study, the ‘Born in Bradford’ cohort, and examine international legal frameworks from Norway and Sweden to understand recent changes in international governmental approaches. The discussion will examine the policy landscape in England and Wales, including legislative attempts to regulate or ban such marriages, the vital role of genetic counselling, and the multifaceted public health arguments both for and against legal intervention. This article aims to explore the delicate equilibrium that must be struck between respecting individual autonomy and cultural practices and fulfilling the state’s crucial responsibility to protect public health.

## Understanding Genetic Risk in Consanguineous Unions

Building upon the foundational understanding of consanguinity, a deeper grasp of genetic principles is essential to comprehend the health implications of such unions. Human traits are governed by genes, each possessing various allelic forms, with individuals inheriting two copies of each gene—one from each parent. Autosomal recessive genetic disorders manifest when an individual inherits two identical copies of a faulty allele. In consanguineous unions, the shared ancestry between spouses significantly increases the probability of their offspring inheriting two identical copies of a recessive allele from a common ancestor. This phenomenon is quantified by the ‘coefficient of inbreeding,’ which represents the probability that an individual inherits two copies of the same gene from a common ancestor. For first-cousin offspring, this coefficient is 1/16, signifying a 1 in 16 chance that any given gene pair is identical by descent. For second-cousin offspring, the coefficient of inbreeding is lower at 1/64, indicating a reduced, though still elevated, risk compared to the general population.^1^ This illustrates a spectrum of risk: the closer the biological relationship between parents, the higher the coefficient of inbreeding and, consequently, the greater the likelihood of offspring inheriting two copies of a recessive allele.

This heightened homozygosity directly correlates with an increased likelihood of adverse health outcomes for offspring. This includes a higher risk of birth defects, ranging from minor anomalies to severe, life-limiting conditions such as congenital heart defects, neural tube defects, limb abnormalities, and craniofacial malformations [22, 34, 46]. The prevalence and specific types of these conditions are influenced by the population’s genetic background and the frequency of particular recessive alleles within that group [8]. Furthermore, there is an elevated incidence of autosomal recessive genetic disorders, including Cystic Fibrosis, Beta-Thalassemia, Phenylketonuria (PKU), and Spinal Muscular Atrophy (SMA), alongside various forms of intellectual disability and sensory impairments. Studies consistently demonstrate an increased risk of infant and childhood mortality in offspring of consanguineous unions, often directly attributable to these severe genetic conditions [28, 41, 44, 45].

While the child of an unrelated couple faces a 2–3 percent risk of inheriting a serious genetic disorder and the child of one-time first cousins a 4–6 percent risk—approximately doubling the risk and roughly equating to the risk faced by children of mothers aged over 34 years [41]—these commonly cited figures do not account for the significantly greater risks faced by cousin-parents from endogamous communities. Endogamy refers to the practice of marrying exclusively within a specific ethnic, religious, or social group. When such communities also have a high prevalence of consanguineous unions across multiple generations, the cumulative effect is a substantial reduction in genetic diversity within the group [21]. This multi-generational relatedness means that individuals within these communities are, on average, more genetically similar to each other than individuals in outbred populations, even if they are not first cousins. Consequently, the baseline risk of inheriting recessive disorders is elevated for all members, and for those who also engage in consanguineous marriages, the risk for their offspring can be compounded. In such cases, offspring can have a greater than 10 percent chance of inheriting a serious disorder, representing three or more times the standard risk [40]. It is important to acknowledge that most children born to consanguineous parents will be born without genetic disorders and congenital anomalies.

### Consanguinity and Eugenics: A Historical Perspective

The discussion surrounding consanguineous marriage, particularly in relation to public health, has historical roots that intersect with the eugenics movement of the 19th and early twentieth centuries. Eugenicists, driven by the belief in improving the human gene pool, often targeted consanguineous unions as a source of ‘undesirable’ hereditary traits. They argued that the increased likelihood of offspring inheriting two copies of faulty recessive alleles from common ancestors contributed to a perceived ‘degeneration’ of the population, thereby justifying interventions to discourage or prohibit such marriages [35]. This historical perspective is critical, as it highlights how scientific and medical discourse, even when nascent, could be co-opted to support social and political agendas. Paul and Spencer, demonstrate how concerns about consanguinity were woven into broader societal anxieties about heredity and national health. They illustrate that even in contexts where explicit eugenics movements were not dominant, the underlying rationale for regulating cousin marriage often mirrored eugenic ideals, focusing on preventing the transmission of perceived genetic defects for the betterment of society [35].

Recognising this historical backdrop is vital for contemporary policy discussions. While current arguments for regulating consanguineous marriage centre on public health imperatives, the historical parallels are hard to ignore. The present challenge for policymakers is to address legitimate public health concerns through carefully considered, evidence-based strategies that actively avoid stigmatising or discriminating against specific communities, and which consistently uphold human rights, so as to prevent any inadvertent alignment with past eugenic justifications.

## The ‘Born in Bradford’ Study

The ‘Born in Bradford’ (BiB) study is one of the most significant and comprehensive investigations into the health outcomes of children born in a multi-ethnic, largely urban setting, with a particular focus on the impact of consanguineous marriage [27, 37]. Its findings have profoundly shaped the understanding and policy discussions surrounding consanguinity in England and Wales and internationally. The study was conducted in Bradford, West Yorkshire, England, a city with a substantial South Asian population, predominantly of Pakistani origin, where consanguineous unions are prevalent. It aimed to provide robust, evidence-based insights into local health disparities, moving beyond anecdotal evidence. Researchers followed over 13,500 babies born between 2007 and 2011, alongside their parents, collecting data through detailed antenatal surveys, linking with National Health Service (NHS) health records, and analysing biological samples.

The study revealed crucial demographic and health-related findings. Approximately 37% of babies in the Bradford cohort were born to related parents, with first-cousin marriages being the most common type. This figure was particularly striking within the Pakistani-origin population, where over 60% of marriages were found to be consanguineous. More significantly, the BiB study provided statistically compelling evidence linking consanguineous marriage to increased health risks for offspring. Children from these unions faced nearly double the risk of congenital anomalies compared to those from non-consanguineous parents, rising from a background risk of around 3% to 6%. Additionally, the study identified a higher prevalence of rare genetic disorders and learning disabilities, aligning with the genetic principles of increased homozygosity for recessive alleles [44]. Consistent with broader research, higher infant and childhood mortality rates were also confirmed among children of consanguineous parents, often attributed to severe genetic conditions. One of the most impactful contributions of BiB was its estimation of attributable risk: despite accounting for a minority of births, consanguineous marriages were responsible for approximately 30% of all congenital anomalies and 25% of infant mortality in Bradford. This demonstrates a significant public health burden extending beyond individual families. While the study’s findings are specific to the Bradford population, they provide a powerful dataset that has been pivotal in informing public health campaigns, genetic screening discussions, and crucial legal and policy debates around intervention in the UK more broadly.

## Legal and Social Responses in Europe

The legal landscape surrounding consanguineous marriage varies significantly across Europe, reflecting diverse cultural norms, historical contexts, and differing interpretations of individual rights versus public health interests [30]. Examining the approaches in Norway and Sweden provides a valuable contrast to the position in England and Wales, highlighting different legal philosophies.

The selection of Norway and Sweden as primary comparators to the UK is deliberate and strategic. Both are Western European democracies with robust welfare states and strong commitments to human rights, making their legal and social contexts highly relevant to discussions in England and Wales. Crucially, both nations have recently undertaken or are in the process of implementing explicit legislative shifts from a historically permissive stance on first-cousin marriage to one of outright prohibition. This recent, decisive movement, driven by a combination of public health concerns (specifically the genetic implications for offspring) and efforts to combat forced marriage, provides a timely and direct parallel to the current policy debate in the UK, particularly concerning Richard Holden MP’s proposed Bill. The shared rationale and recent legislative actions make Norway and Sweden particularly pertinent examples for understanding the evolving governmental approaches to this complex issue within a similar socio-political framework.

### Nordic Marriage Laws: The Evolving Stance on Cousin Marriage in Norway and Sweden

Both Norway and Sweden have historically maintained a permissive stance towards first-cousin marriages, largely prioritising individual liberty, free will, and consent in marital choices, with legal frameworks focusing on aspects such as age and capacity rather than genetic relatedness. However, recent legislative developments in both countries signify a decisive shift towards prohibiting such unions, driven by similar public health and human rights concerns.^2^

#### Legislative Shifts and Rationale for Prohibition

Norway implemented legislation to ban cousin marriages entirely as of September 2023. This marks a significant intervention. The Norwegian Tax Administration’s guidelines now reflect this legislative reality, confirming that first-cousin marriages are no longer legally recognised [6]. For marriages entered into overseas after January 1, 2025, if one of the parties is Norwegian or was residing in Norway at the time of marriage, consanguineous unions will generally not be recognised. Entering a consanguineous marriage itself, if there is no evidence of force or other criminal acts, will not lead to criminal prosecution.

Sweden is also enacting a similar ban on cousin marriages, which is expected to come into effect on July 1, 2026. This decision follows a special commission’s final report in October 2024, which recommended the prohibition of marriage between first cousins and other close relatives, and that Sweden would no longer recognise such marriages occurring abroad [34].

The primary drivers for these legislative changes in both nations are consistent: A stronger emphasis on public health and child welfare, particularly in relation to children’s health stemming from the genetic implications of consanguinity. Proponents argue that the state has a legitimate interest and duty to intervene when a practice demonstrably increases the risk of severe health conditions for offspring, aligning with the *parens patriae* principle. Additionally, governments in both countries believe that the ban will help combat forced marriages, as some consanguineous unions have been linked to such practices.

This convergence highlights a regional trend in Scandinavian public health policy, reflecting a growing consensus that the demonstrated genetic risks and potential for forced marriage outweigh traditional arguments for individual autonomy in this specific context.

### England and Wales: Marriage Laws and Cousin Marriage

The legal position on cousin marriage in England and Wales remains permissive and consanguineous marriages are still legally recognised [31]. The legal framework governing marriage prohibitions has a complex history, largely rooted in ecclesiastical law, which for centuries dictated who could and could not marry. While marriage between cousins, including first cousins, has historically always been permitted, prohibitions extended to certain relationships by affinity i.e. marriage to a relative of a deceased spouse, even where no blood relationship existed. For example, before the Marriage Act of 1907 [25], a man could not legally marry his deceased wife’s sister. The comprehensive list of forbidden marriages, drawn up by the Church of England in 1560 and printed in The Book of Common Prayer of 1662 [47], remained largely unchanged for nearly 350 years. This list extensively detailed prohibitions for both men and women marrying direct lineal ascendants or descendants, siblings, and various in-laws.

The early twentieth century marked a significant shift in attitudes towards marriage prohibitions, leading to a relaxation of previously stringent rules in England and Wales. This modernisation began with a series of legislative changes, consolidated and extended by the Marriage Act 1949, and further refined by the Marriage Act 1986, which now forms the foundation of the current legal framework [24]. Under this Act, marriage remains prohibited between certain direct blood relatives, specifically a man and his mother, daughter, grandmother, granddaughter, sister, half-sister, aunt, half-aunt, niece, or half-niece. Correspondingly, a woman may not marry her father, son, grandfather, grandson, brother, half-brother, uncle, half-uncle, nephew, or half-nephew.^3^ This permissive legal stance rooted in its unique legal history, places it in a different position from the evolving Scandinavian approach, which increasingly opts for legal prohibitions based on public health and child welfare concerns. The varying approaches underscore the complex interplay between historical legal traditions, human rights principles, and contemporary public health concerns across different jurisdictions, and highlights the ongoing debate in the England and Wales regarding whether to follow the Scandinavian example [40, 41].

## Legal Recognition: The Consequences of a Legally Valid Marriage

A crucial aspect of this debate, often overlooked, is the legal significance of a marriage being formally recognised. A legally valid marriage in England and Wales confers a distinct set of rights and responsibilities that do not automatically apply to unrecognised unions, even if children are involved. The legal system in England and Wales separates the status of a spouse from the status of a parent, ensuring that a child’s welfare is protected regardless of their parents’ marital status. However, the legal protections and obligations for the adults themselves differ considerably.

For spouses in a legally recognised marriage, these rights include both financial and property rights. Spouses have specific legal rights to property and assets acquired during the marriage. In the event of a divorce or death, the division of marital property is governed by matrimonial law, which often provides more substantial protections for a financially weaker spouse.^4^ A surviving spouse is typically entitled to a share of their deceased partner’s estate under intestacy rules (where there is no will).^5^ This contrasts with unmarried partners, who have no automatic right to inherit from their partner’s estate. Spouses can have specific entitlements to a partner’s pension and other state benefits, such as bereavement support payment, which are not available to unmarried partners.

Marriage provides a legal framework for a couple’s relationship, offering protection from domestic abuse and other forms of exploitation through specific legal remedies. However, for couples in unrecognised unions, often referred to as ‘common-law’ marriages, these rights do not exist. While both parents, regardless of marital status, have legal responsibilities toward their children, such as parental responsibility and child maintenance, the adults themselves do not have the same legal protections. For a common-law spouse, the lack of legal recognition can leave one partner financially vulnerable upon separation or death.

Legally prohibiting marriage between first cousins would render such unions void in England and Wales. While couples from communities with a history of this practice may still hold a ceremony, their union would lack legal recognition. This absence of legal status would deny them the rights and protections afforded by marriage, which could lead to financial instability and social vulnerability. This impact would be particularly severe for women and children, who are often the most exposed to such risks.

## England and Wales: Policy Debates and Public Health Arguments

In England and Wales this longstanding legal position regarding first-cousin marriage has faced increasing scrutiny, particularly considering recent public health data [4, 37]. The most prominent recent attempt to challenge the legal status quo came from Richard Holden MP. In December 2023, then a Conservative MP for Northwest Durham, Holden announced his intention to introduce a Private Members’ Bill [26] aiming to address the issue of cousin marriage. His initiative stems from growing concerns about the associated health risks and the perceived strain on the NHS.

Relying on statistics from the ‘Born in Bradford,’ study [17], Holden argues that the state has a responsibility to mitigate these preventable health risks and that the current legal silence on the matter is inadequate. While his Bill primarily addresses genetic health and NHS strain, the broader discourse around consanguineous unions in England and Wales also encompasses concerns about individual autonomy and the prevention of forced marriage, which are seen as critical public health and safeguarding issues. His legislative intent is to bring the legal framework into line with what he perceives as a modern public health imperative, aiming to reduce suffering and alleviate the economic burden on the NHS. Holden argues that if the state had an interest in other areas of public health, such as smoking or obesity, it should also address preventable genetic conditions. The government’s own policy documents, such as those from the Department of Health and Social Care, often emphasise preventative health and reducing health inequalities, providing a broad context for such legislative ambition [9].

The proposed Marriage (Prohibited Degrees of Relationship) Bill 2025 is a concise legislative instrument that will make specific amendments to the Marriage Act 1949. Its core objective lies in Clause 1(2), inserting ‘first cousin’ into Schedule 1, sub-paragraph 1(1) of the 1949 Act, thereby statutorily rendering such marriages legally void. To ensure clarity and prevent ambiguity, Clause 1(3) precisely defines ‘first cousin’ as ‘the child of a parent’s sibling,’^6^ and it is designed to become effective three months after receiving Royal Assent, allowing for a brief period of transition. The legislation would be prospective and would not nullify existing marriages.

From a public health perspective, the Bill is framed as a preventative measure designed to reduce the incidence of preventable illness and disability, and to mitigate the significant economic and resource strain on the NHS that arises from managing these conditions [36]. This includes expenses for specialist medical care like frequent hospitalisations, complex surgeries, and ongoing treatment for conditions such as congenital heart defects or severe metabolic disorders. Additionally, there are considerable costs for long-term care, therapies, such as, physiotherapy, occupational therapy and speech therapy; and social support services, all of which draw on public funds. The core argument here is that the state’s duty to protect public health and ensure the efficient allocation of finite healthcare resources fully justifies legislative intervention, even in matters traditionally considered within the realm of personal choice. If a practice demonstrably contributes to preventable disease and disability, the state’s responsibility to intervene is activated. This resonates with the public health objectives outlined in national health strategies and legislation, such as the Health and Social Care Act 2012, which emphasises public health duties. This aligns seamlessly with broader governmental policy emphasising preventative health and reducing health inequalities [9].

However, despite a compelling public health rationale, the Bill is likely to face considerable legal and ethical challenge, primarily grounded in fundamental human rights principles. A direct prohibition on first-cousin marriage would likely be challenged as an infringement on the fundamental right to marry, as enshrined in Article 12 of the European Convention on Human Rights (ECHR) [14, 39].While this right is not absolute and may be subject to national laws, any interference must be ‘prescribed by law,’ pursue a ‘legitimate aim,’ and crucially, be ‘necessary in a democratic society’ and ‘proportionate’ to that aim. Opponents to the Bill will certainly argue that an outright ban constitutes a disproportionate measure, particularly given that many consanguineous marriages might be regarded as consensual^7^ and that less restrictive alternatives, such as comprehensive genetic counselling, are available [15].

The jurisprudence of the European Court of Human Rights, exemplified by cases like *Sheffield and Horsham v United Kingdom* [42],^8^ consistently sets a high bar for state interference with marital and family life. Given that the choice of a marital partner is a profoundly personal decision, inherently covered by Article 8 ECHR’s guarantee of respect for private and family life [13], any legal prohibition would constitute a significant and potentially unwarranted infringement upon this private sphere, necessitating robust justification. A ban on consanguineous marriage could drive the practice underground. In communities where consanguineous unions are embedded culturally or religiously, a legal prohibition would not necessarily eliminate the practice but rather push it into clandestine forms. This could involve informal unions not recognised by the state, or marriages conducted abroad and not registered domestically, making it harder to monitor or address related issues. This could lead to further alienation of consanguineous parents from seeking genetic counselling or other healthcare. The fear of judgment, legal repercussions, or being socially ostracised could lead them to avoid prenatal screening, genetic counselling, and other vital health services that could identify and manage risks associated with consanguinity, thereby undermining the very public health goals a ban aims to achieve [19]. Furthermore, while the Bill’s primary aim is genetic health, concerns about forced marriage and the protection of individual autonomy against coercion also form a significant part of the broader public health and human rights debate surrounding consanguineous unions. The potential for such a ban to create a climate of fear and distrust among those in consanguineous relationships would be a counterproductive outcome, hindering efforts to promote health and well-being within these communities.

However, internationally, human rights provisions are highly qualified, affording signatory states a broad ‘margin of appreciation’ on contentious issues. As a result, a ban on consanguineous marriages is likely to be upheld if it can be justified on any logical, rational or practical ground [33]. This position is further reinforced by the fact that multiple Eastern European countries, and now including Norway and Sweden, already implement outright or partial bans on cousin marriage. In *Theodorou and Tsotsorou v Greece* [2]^9^ it was noted that ‘as a general rule, the limitations that affect the ability to enter into marriage [concerning issues of] consent, consanguinity or prevention of bigamy are likely to be compatible with Article 12’ [43].^10^ Moreover, numerous democratic East Asian nations and many US states [29]^11^ impose restrictions or outright bans on cousin marriage, which have consistently withstood constitutional challenge over time [38]. Consequently, any legal challenge against such restrictions could very well fail. A closer examination of multiple Eastern European countries reveals a historical and often continuing legal stance that prohibits or restricts cousin marriage, primarily influenced by long-standing ecclesiastical law, particularly that of the Orthodox Church. These prohibitions, deeply embedded in the legal and social fabric, often predate modern public health concerns and are rooted in broader societal and religious disapproval of unions deemed too close. This historical context underscores that legal restrictions on consanguinity are not a novel concept in Europe and have long been considered compatible with societal norms and, by extension, human rights frameworks [6].

Critics would also contend that while the Bill might appear neutral, its practical effect would disproportionately impact specific ethnic and religious communities in England and Wales where consanguineous marriage is culturally prevalent [20]. This raises serious concerns about indirect discrimination, potentially violating Article 14 ECHR when read in conjunction with Articles 8 or 12 [12]. Indirect discrimination occurs when a seemingly neutral provision, criterion, or practice puts individuals of a particular racial or ethnic origin at a particular disadvantage compared with others, unless that provision, criterion, or practice is objectively justified by a legitimate aim and the means of achieving that aim are appropriate and necessary. Given that consanguineous marriage is significantly more common within certain South Asian communities in the UK, a blanket ban, while not explicitly targeting these groups, would disproportionately affect their ability to marry according to their cultural practices. This could lead to a sense of stigmatisation and marginalisation, implying that their cultural practices are inherently problematic or undesirable, thereby exacerbating existing social inequalities [7, 19, 32].

The Marriage (Prohibited Degrees of Relationship) Bill 2025, if enacted, would fundamentally alter marriage law in England and Wales by prohibiting first-cousin marriages. While driven by legitimate public health concerns and a desire to mitigate preventable genetic disorders and NHS strain, its passage would inevitably invite legal challenge on human rights grounds, particularly concerning the rights to marry, private and family life, and non-discrimination. The Bill represents a critical point in the ongoing debate about the appropriate balance between state intervention for public health and the protection of individual and cultural freedoms within a democratic society. Its ultimate fate would depend on parliamentary will and its ability to withstand scrutiny under the Human Rights Act 1998.

## Alternatives to Banning Cousin Marriage

Instead of outright prohibition, more ethically sound public health strategies are available to address the health implications of consanguineous marriages. Banning such unions erode individual autonomy and the right to informed decision-making, rather than empowering individuals through education and support. They risk condemning cultural practices outright, potentially alienating communities and undermining trust, which is crucial for effective public health engagement. Furthermore, a ban could inadvertently conflate consensual consanguineous unions with the distinct and serious issues of forced marriage and abuse, failing to address these complex safeguarding concerns directly and sensitively.

Genetic counselling services could improve by becoming more accessible [3], ensuring that comprehensive and non-directive information about genetic risks, inheritance patterns, and screening/diagnostic options is readily available to prospective parents. The goal is to empower individuals to make autonomous and informed decisions [10]. Within tightly knit endogamous communities, there is mounting awareness regarding the prevention of congenital and genetic disorders in offspring, and this is driving an increasing number of couples who are contemplating marriage and reproduction to seek genetic counselling and testing [16]. For couples in consanguineous marriages facing genetic issues, IVF with Preimplantation Genetic Testing (PGT) is increasingly used to screen embryos for disorders, reducing transmission risk and improving pregnancy outcomes. While growing, its specific prevalence among these couples is not widely quantified. However, the use of donor gametes (sperm or egg) presents significant cultural and religious hurdles. Many faiths forbid third-party gamete donation due to concerns about lineage and the sanctity of marriage, making it far less accepted than IVF with PGT using the couple’s own gametes [1].

Furthermore, targeted public health education campaigns are crucial. These initiatives must be culturally sensitive, linguistically appropriate, and disseminated through trusted community channels. The focus should be on raising awareness of genetic risks and promoting informed choices, moving away from condemnation of the practice itself. This approach, by harnessing information sharing without coercion, is akin to successful national screening programmes for conditions like Sickle Cell and Thalassemia. These programmes have demonstrated that providing clear, accessible information about carrier status and inheritance patterns, coupled with support for reproductive choices, can significantly improve health outcomes within at-risk communities through voluntary participation and informed decision-making, rather than through legislative bans.^12^ Such campaigns build trust and empower individuals to make choices aligned with their health goals and cultural values.

Finally, proactive genetic screening programmes for common recessive conditions in at-risk populations, alongside robust community engagement, can significantly mitigate risks [5]. Identifying carrier status before marriage allows couples to make informed decisions and explore options. By collaborating with community and faith leaders, and improving health literacy, these strategies foster trust and facilitate voluntary engagement, ultimately promoting better health outcomes [11]. Community and faith leaders are invaluable intermediaries; their trusted positions allow them to legitimise health messages, facilitate open dialogue, and help bridge potential cultural or communication gaps between health authorities and community members. Improving health literacy goes beyond simply providing information; it involves ensuring that individuals can understand, interpret, and apply complex health information to make informed decisions about their own health and reproductive choices. This requires clear, jargon-free communication, culturally relevant materials, and opportunities for interactive learning, all of which contribute to sustainable behavioural change and greater individual autonomy.

### Addressing Forced Marriage and Abuse through Public Health Strategies

It is important to clarify that while strategies like genetic counselling, public health education, and proactive genetic screening are highly effective in empowering individuals to make informed choices regarding genetic health risks, they are less directly effective in preventing forced marriage or abuse. It’s crucial to recognise that concerns in nations like Norway and Sweden, as well as in the broader UK discourse, extend beyond genetic health risks and associated healthcare costs. These nations have explicitly cited the prevention of forced marriage and the combating of abuse as significant drivers for legislative change, viewing these issues as critical public health matters impacting individual autonomy, mental well-being, and physical safety. While Richard Holden MP’s proposed Marriage (Prohibited Degrees of Relationship) Bill 2025 in England primarily focuses on mitigating the economic burden on the NHS, the wider public health discussion around consanguineous unions in the UK often encompasses the serious issues of forced marriage and abuse. These concerns are particularly salient within certain communities where consanguineous marriage is culturally prevalent. Data from the Forced Marriage Unit (FMU) illustrates that a considerable proportion of forced marriage cases involve individuals from South Asian backgrounds, where consanguineous unions may sometimes be a factor. However, it is vital to stress that consanguinity itself does not equate to forced marriage. The FMU provided advice or support in 802 cases of forced marriage in 2023, with Pakistan, Bangladesh, Afghanistan, and India being among the top countries of origin [18], underscoring the ongoing relevance of forced marriage as a public health and safeguarding concern in the English context.

The prevention of forced marriage and abuse requires a distinct, albeit complementary, set of public health and safeguarding interventions. These interventions include robust legal frameworks and enforcement, such as the Forced Marriage (Civil Protection) Act 2007 and the Anti-social Behaviour, Crime and Policing Act 2014, which criminalise forced marriage and provide civil protection orders. Additionally, providing accessible and culturally sensitive specialised support services for victims or those at risk—including helplines, refuges, and counselling—is crucial for safeguarding individuals’ physical and psychological well-being. Community engagement and awareness initiatives are also vital for promoting understanding about individual rights, the illegality of forced marriage, and available support. This often involves collaboration with community leaders, schools, and religious institutions to challenge harmful practices. Furthermore, professional training for police, social workers, and healthcare providers is essential to equip them with the knowledge and skills to effectively identify, respond to, and prevent forced marriage and abuse. A multi-faceted approach, which combines genetic health strategies with dedicated anti-forced marriage initiatives, is necessary to address the full spectrum of public health concerns associated with consanguineous unions.

## Conclusion

The debate surrounding consanguineous marriage highlights a critical tension between public health concerns and fundamental human rights. Despite clear evidence from studies like ‘Born in Bradford’ highlighting increased genetic risks that burden the NHS, along with serious concerns about forced marriage and individual autonomy, a complete legal ban on consanguineous unions would present considerable difficulties. International precedent offers a mixed picture, with some nations moving towards bans while others, like England and Wales, prioritising individual autonomy, albeit with evolving perspectives.

Legislative attempts, such as Richard Holden MP’s Bill, have brought these public health arguments to the fore. However, these proposals face strong counterarguments rooted in the right to marry, privacy, and non-discrimination, alongside concerns about stigmatisation and the potential for driving the practice underground [23]. The core dilemma lies in balancing the state’s role in safeguarding well-being, particularly for children, against deeply held principles of liberty and cultural freedom.

Moving forward, the focus must shift from punitive legal measures to nuanced, culturally sensitive public health strategies. Empowering individuals and communities through comprehensive, accessible, and non-directive genetic services is key. This includes targeted public health education, enhanced genetic screening, and robust genetic counselling. Such approaches respect human rights by offering informed choice, leading to more sustainable improvements in public health outcomes without legal coercion.

## Data Availability

No datasets were generated or analysed during the current study.

## Footnotes

1 Children from non-consanguineous unions face a background risk of around 3% for congenital anomalies. This risk is approximately doubled to 6% for children born to first-cousin parents.

2 Norway. *Lov om ekteskap (ekteskapsloven)* [Marriage Act] of July 4, 1991, No. 47, last consolidated June 15, 2018, with amendments concerning the prohibition on marriage between close relatives incorporated as of January 7, 2025. In Sweden on October 8, 2024, a special commission appointed by the Swedish government to consider bans on marriage between cousins and other close relatives published its final report—*F rbud mot äktenskap mellan kusiner och vissa andra nära släktingar* Ds 2024:20 (Prohibition of marriage between cousins and certain other close relatives Ds 2024:20). Under the new rules, marriage between first cousins would be prohibited, and Sweden would no longer recognise such marriages occurring abroad. The possibility to apply for an exception to marry a sibling with whom one shares one parent (known in Sweden as half-siblings) would also be removed. If adopted, the new law would enter into force on July 1, 2026.

3 Under the Marriage (Prohibited Degrees of Relationship) Act 1986, the prohibition on marriage between direct blood relatives also applies to same-sex marriages. While the language of the statute is gendered, the underlying principle is that marriage is forbidden between individuals who are direct blood relatives in those specified categories, regardless of their gender. The focus of the legislation is on the familial relationship by blood, not the gender of the individuals involved.

4 Section 25, Matrimonial Causes Act 1973.

5 Inheritance and Trustees’ Powers Act 2014.

6 The Bill’s geographical scope is confined to England and Wales and if passed, will be formally cited as the Marriage (Prohibited Degrees of Relationship) Act 2025.

7 Clear data on the consensual nature of a consanguineous marriage is sparse. Richard Holden MP recently stated: ‘Some critics say a ban would infringe upon people’s freedom—but what freedom are we protecting? The reality for so many is a life predetermined by bloodline and birth order. We are not protecting a freedom; we are perpetuating oppression. Whose freedom, if any, are we protecting? Purely the freedom of the oppressor to oppress and keep down—not the freedom of the individual. The state already intervenes where power dynamics distort consent. We rightly outlaw relationships between teachers and pupils or therapists and clients, because of the imbalance.’ Richard Holden MP, HC Deb [Hansard] W 18 June 2025, vol 769, col 112WH.

8 *Sheffield and Horsham v United Kingdom* (2003) 37 EHRR 31. The European Court of Human Rights (ECtHR) views the choice of a marital partner as a *core* aspect of private and family life, protected under Article 8 of the European Convention on Human Rights (ECHR).

9 (App no 57854/15) [2019] ECHR 611, para 28).

10 Consistent with prevailing legal norms, the European Court of Human Rights (ECtHR) addressed the legitimacy of the consanguinity bar in cases of sibling incest in *Stübing v. Germany* (App. No. 43547/08, judgment of 12 April 2012). The Court underscored the widespread societal and legal disapproval, noting a ‘broad consensus transpires that sexual relationships between siblings are neither accepted by the legal order nor by society as a whole,’ regardless of whether criminal sanctions are imposed (para. 61). The ECtHR further acknowledged the German Federal Constitutional Court’s rationale for criminalisation, which invoked the protection of family integrity, sexual self-determination, and public health, positing that ‘sexual relations between siblings might seriously damage family structures and consequently society as a whole’ (para. 63).

11 National Conference of Commissioners on Uniform State Laws. (1970). *Handbook of the National Conference of Commissioners on Uniform State Laws and proceedings of the annual conference meeting* (Vol. 79, pp. 1, 186–187). This publication includes discussions on striking first cousins from the model Uniform Marriage and Divorce Act to align with ‘the recent legislative trend toward permitting first cousin marriages.’.

12 *NHS launches lifesaving sickle cell campaign*. Available online: https://www.england.nhs.uk/2022/06/nhs-launches-lifesaving-sickle-cell-campaign/ (accessed on 24 July 2025).
